# Evolutionary insights into umami, sweet, and bitter taste receptors in amphibians

**DOI:** 10.1002/ece3.8398

**Published:** 2021-12-03

**Authors:** Huaming Zhong, Jie Huang, Shuai Shang, Baodong Yuan

**Affiliations:** ^1^ College of Biology and Food Shangqiu Normal University Shangqiu China; ^2^ College of Biological and Environmental Engineering Binzhou University Binzhou China

**Keywords:** amphibian, anurans, bitter taste receptor, caecilians, umami/sweet taste receptor

## Abstract

Umami and sweet sensations provide animals with important dietary information for detecting and consuming nutrients, whereas bitter sensation helps animals avoid potentially toxic or harmful substances. Enormous progress has been made toward animal sweet/umami taste receptor (Tas1r) and bitter taste receptor (Tas2r). However, information about amphibians is mainly scarce. This study attempted to delineate the repertoire of *Tas1r*/*Tas2r* genes by searching for currently available genome sequences in 14 amphibian species. This study identified 16 *Tas1r1*, 9 *Tas1r2*, and 9 *Tas1r3* genes to be intact and another 17 *Tas1r* genes to be pseudogenes or absent in the 14 amphibians. According to the functional prediction of *Tas1r* genes, two species have lost sweet sensation and seven species have lost both umami and sweet sensations. Anurans possessed a large number of intact *Tas2rs*, ranging from 39 to 178. In contrast, caecilians possessed a contractive bitter taste repertoire, ranging from 4 to 19. Phylogenetic and reconciling analysis revealed that the repertoire of amphibian *Tas1rs* and *Tas2rs* was shaped by massive gene duplications and losses. No correlation was found between feeding preferences and the evolution of *Tas1rs* in amphibians. However, the expansion of *Tas2rs* may help amphibians adapt to both aquatic and terrestrial habitats. Bitter detection may have played an important role in the evolutionary adaptation of vertebrates in the transition from water to land.

## Graphical abstract

Amphibians have the largest number of Tas2rs across vertebrates. The repertoire of amphibian Tas1rs and Tas2rs was shaped by massive gene duplications and losses. Bitter detection played an important role in the evolutionary adaptation of vertebrates in the transition from water to land.

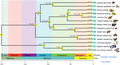

## 1 INTRODUCTION

Taste (or gustation) is one of the primary mechanisms that animals use to assess the nutritional quality of foods. Vertebrates can perceive five fundamental taste modalities: bitter, umami, sweet, salty, and sour (Kinnamon & Cummings, 1992; Lindemann, 1996, 2000; Stewart et al., 1997; Yarmolinsky et al., 2009). Each taste sense is thought to have evolved to face a challenge or play a specific role in species evolution (Marco & Davide, 2017; Peng et al., 2020). For instance, sweet and umami tastes are associated with the intake of nutrients such as carbohydrates and protein. Conversely, bitter detection prevents animals from ingesting potentially poisonous foods (Herness & Gilbertson, 1999). Sweet, umami and bitter substances are detected by type II taste receptor cells (TRCs) that function through G protein‐coupled receptors (GPCRs; Adler et al., 2000).

Sweet and umami taste are activated by taste 1 receptors (Tas1r), including Tas1r1, Tas1r2 and Tas1r3 (Bufe & Meyerhof, 2006; Li et al., 2002; Temussi, 2006). They are encoded by *Tas1r1*, *Tas1r2*, and *Tas1r3* genes, respectively. Each *Tas1r* contains a GPCR‐like domain and a large N‐terminal extracellular domain (Chen et al., 2009). Tas1r3 is co‐expressed with either Tas1r1 or Tas1r2, which form into heterodimers: a Tas1r1‐Tas1r3 heterodimer functions as an umami taste receptor, whereas a Tas1r2‐Tas1r3 heterodimer senses sweet compound (Li et al., 2002; Nelson et al., 2001). Although most vertebrates have three complete *Tas1r* genes, the repertoires of *Tas1rs* in other species may differ in both sequence and numbers. Three main processes have shaped the different numbers of *Tas1rs* in different species: gene loss, pseudogenization, and duplication (Liu et al., 2014). For example, *Tas1r1* is a pseudogene in the giant panda (*Ailuropoda melanoleuca*; Zhao, Yang, et al., 2010) and six pinniped species (Sato & Wolsan, 2012). *Tas1r1* is absent, unamplified, or pseudogenized in 31 species of bats examined (Zhao et al., 2012). *Tas1r2* is lost in the genome of the zebra finch (*Taeniopygia guttata*) and chicken (*Gallus gallus*) and pseudogenized in some carnivore species (Jiang et al., 2012; Li et al., 2005, 2009; Zhao, Zhou, et al., 2010). In reptiles, *Tas1r* genes possibly have been lost or pseudogenized in snakes; in testudines and crocodilians, *Tas1r* genes are either intact or partial (Feng & Liang, 2018). These mutations are thought to result in the disfunction of *Tas1r* and then affect sweet/umami recognition in animals. Nevertheless, a lineage‐specific increase in the number of *Tas1rs* has been described in fish. In 15 fish species examined, the number of *Tas1r2* genes differs widely, ranging from 1 to 4 (Dong et al., 2018). To date, several studies have investigated the ecological factors that drive the evolution history of *Tas1rs* in vertebrates. The evolution of *Tas1r* genes is sometimes explained by feeding ecology (Zhao, Zhou, et al., 2010), sometimes inconsistent with dietary differences (Feng & Liang, 2018; Feng & Zhao, 2013; Zhao et al., 2012; Zhao & Zhang, 2012).

Bitter substances are recognized by taste 2 receptors encoded by the *Tas2r* gene family (Adler et al., 2000). *Tas2r* genes possess a GPCR‐like domain and a short extracellular N‐terminus. They are ~900 bp and lack introns. Several studies have indicated that the repertoire of *Tas2rs* showed a very dynamic evolution among species. For instance, the number of intact (functional) *Tas2rs* is subject to intense variation: 0 in cetaceans and penguins (Feng et al., 2014; Zhu et al., 2014), 25 in humans (Go et al., 2005; Shi et al., 2003), 3–50 in lizards (Zhong et al., 2017, 2019), 1–5 in teleost (Dong et al., 2018), and 80 in the coelacanth (*Latimeria chalumnae*; Syed & Korsching, 2014). Not surprisingly, there are varying numbers of pseudogenes among species. Pseudogenization has occurred in almost all *Tas2rs* in cetaceans (Feng et al., 2014; Zhu et al., 2014).

Numerous studies have attempted to explain the evolutionary and ecological significance of *Tas2r*s. It is generally accepted that the taste receptor gene family has undergone a complex evolutionary process. They are susceptible to gene duplication, gene deletion, pseudogenization, positive selection, and other factors, resulting in the expansion or contraction of specific gene families among different evolution branches (Dong et al., 2009; Go, 2006; Hayakawa et al., 2014; Li & Zhang, 2014; Shi et al., 2003; Wang & Zhao, 2015). This complexity is assumed to reflect the evolutionary needs of the respective species. For instance, the relationship between the *Tas2r* numbers of vertebrates and their corresponding dietary habits has been addressed. Actually, some studies have uncovered the probable correlation between diets and *Tas2r* numbers: herbivores and insectivores who encounter bitter substances more frequently possess more *Tas2rs* than carnivores (Hu & Shi, 2013; Jiang et al., 2012; Liu et al., 2016; Wang & Zhao, 2015; Zhong et al., 2017). These findings have suggested that the dietary toxin content is one of the primary selective forces for the differences in *Tas2rs* repertoires among species.

Other than dietary habits, foraging patterns may also affect the repertoires of *Tas2r* genes. Previous studies have indicated that cetaceans (0–1; Zhu et al., 2014; Feng et al., 2014), snakes (1–2; Zhong et al., 2017), and penguins (0; Zhao et al., 2015) possess a dramatic contraction in the number of functional *Tas2r* genes. Their behavior of swallowing food whole without mastication reduces the contact of the TRCs with bitter stimuli, resulting in less contact with poisonous foods.

So far, studies on the function and adaptive evolution of *Tas1r*/*Tas2r* genes have been carried out extensively in many species. However, data are quite limited in *Tas1r*/*Tas2r* families of amphibians, except for the western clawed frog (Behrens et al., 2014; Go, 2006; Shi & Zhang, 2006). Amphibian is the transition lineage from aquatic lifestyle to a terrestrial one in the history of vertebrate evolution and plays an important role in animal evolution. To gain extensive, systematic, and efficient evolution research on *Tas1r*/*Tas2r*, it was considered worthwhile to investigate more data and adaptive evolution of amphibian species. Therefore, this study aimed to examine the repertoires of *Tas1r*/*Tas2r* genes in amphibians and predict their functionality using available genome assemblies. This study recovered the phylogenetic relationship and determined the duplication and loss events of *Tas2rs* to understand their birth‐and‐death process in this evolutionary group. Furthermore, the selective pressure of *Tas1rs* in amphibians was estimated.

## 2 MATERIALS AND METHODS

### 2.1 Taxonomic sampling of genome data

Class Amphibia is generally classified into three orders: Anura (anurans), Caudata (urodeles), and Gymnophiona (caecilians). This study focused on currently available genomes of 14 species from the National Center for Biotechnology Information (NCBI) databases. They are Leishan spiny toad (*Leptobrachium leishanense*), Mexican spadefoot toad (*Spea multiplicata*), African bullfrog (*Pyxicephalus adspersus*), American bullfrog (*Lithobates catesbeianus*), common frog (*Rana temporaria*), Tibetan Plateau frog (*Nanorana parkeri*), Eastern banjo frog (*Limnodynastes dumerilii*), strawberry poison frog (*Oophaga pumilio*), Asiatic toad (*Bufo gargarizans*), western clawed frog (*Xenopus tropicalis*), and African clawed frog (*Xenopus laevis*) in Anura and two‐lined caecilian (*Rhinatrema bivittatum*), Gaboon caecilian (*Geotrypetes seraphini*), and tiny cayenne caecilian (*Microcaecilia unicolor*) in Gymnophiona. The detailed information of the genome assemblies is provided in Appendix 1. The N50c values of the genomes ranged from 2.9 kb to 20.7 Mb, implying high‐quality assemblies.

### 2.2 Gene annotation

As *Tas2r* genes contain no introns, and most have a similar gene length of ~900 bp, gene annotation was performed by sequence alignment with TBlastN (Altschul et al., 1990), which was implanted in TBtools (Chen et al., 2020). First, previously known Tas2r protein sequences were retrieved from the GenBank or literature and used as initial queries (25 from human, 35 from mouse, 3 from chicken, and 80 from coelacanth). Second, the queries were used to blast against a genome assembly by TBlastN (Altschul et al., 1990), with an *e*‐value of 1 × 10^−10^. BLAST hits of <100 bp were discarded, and the overlapping hits were merged. The remaining records were prolonged for 500 bp in both 5′ and 3′ directions, which were regarded as the genomic locations of the homologous genes. Third, genomic nucleotide sequences were extracted as candidate *Tas2r* genes. The outputs were divided into three categories: intact genes, partial genes, and pseudogenes according to a previous study (Li & Zhang, 2014). Intact genes refer to sequences with >270 amino acids with both start and stop codons. Partial genes refer to sequences that lack either a start or a stop codon. They may be complete genes, but their open reading frames (ORFs) are truncated due to incomplete genome sequencing or assembling. The homology of partial genes in their corresponding genome was analyzed through alignment. If multiple fragment sequences of the same species can be aligned with overlapping regions, they are considered to be from different gene loci. If there are no overlapping regions during alignment, they are considered to be from the same gene site, which may be caused by sequence spacing due to sequencing or assembly. Sequences with premature stop codons and/or ORF‐disrupting mutations were regarded as pseudogenes.

As *Tas1r* genes contain introns, a more complex bioinformatic pipeline was employed. First, previously known Tas1r1, Tas1r2, and Tas1r3 protein sequences were used as queries to identify the genomic locations of homologous genes in a genome. Second, genomic DNA sequences were extracted and used to perform pairwise alignments with query protein sequences by Genewise (Madeira et al., 2019), which provided the exon/intron structures and frameshifting errors. When receiving negative BLAST results, synteny analysis was performed to examine *Tas1r* genes with closely related species as the reference. If neighboring genes flanking *Tas1r* genes could be found, *Tas1r* genes were regarded as absent.

The obtained protein sequences of each Tas1r/Tas2r were verified by the TMHMM method (Krogh et al., 2001) for the presence of seven transmembrane domains. A *Tas1r*/*Tas2r* sequence was regarded as a pseudogene if it did not have seven transmembrane domains. In addition, annotated sequences were examined by reciprocal SmartBLAST (https://blast.ncbi.nlm.nih.gov/blast/smartblast/) as well as phylogenetic analyses to ensure that the best hits are known *Tas1r*/*Tas2r* genes. The gene nomenclature was named with a four‐letter prefix corresponding to the species names as well as a numerical suffix consecutively. For example, the *Tas2r1* gene of *L*. *leishanense* is referred to as Lele_Tas2r1.

### 2.3 Phylogeny of taste receptor genes in amphibians

To explore the evolutionary relationship among *Tas1r*/*Tas2r* genes in amphibians, a phylogenetic analysis of intact genes was performed. Partial genes or pseudogenes were not included in the phylogenetic analysis due to a large number of gap sites after alignments. Multiple sequence alignments of amino acid sequences were performed by MAFFT with the L‐INS‐I strategy (Katoh et al., 2002). GBLOCKS (Castresana, 2000) was then used to optimize the quality of alignment results. The selected conserved region was used in the following analysis. The phylogenetic relationship of the genes was inferred by the maximum likelihood (ML; Dempster, 1977) method. ML phylogenies were inferred using IQ‐TREE (Lam‐Tung et al., 2015) under the model selected by ModelFinder (Kalyaanamoorthy et al., 2017). The branch support analysis was evaluated with 1000 ultrafast bootstraps (Minh et al., 2013). The tree was rooted with vertebrate *V1R*/*V2R* vomeronasal receptor. The procedures processed in ML phylogenies were all implemented in PhyloSuite (Zhang et al., 2020). The visualization of ML tree was performed using iTOL (Letunic & Bork, 1988).

### 2.4 Reconstruction of *Tas2r* repertoire evolution

Large‐scale gene births and deaths are the major forces of functional genetic innovation. To infer the history of births (duplication) and deaths (deletion) of *Tas2r* genes across the amphibian phylogeny, a reconciliation analysis was performed with NOTUNG 2.6 (Chen et al., 2000). This method estimates the history of gene duplication and deletion times by comparing gene tree with species tree. The species tree was estimated from the TimeTree database, which provided the generally accepted phylogenetic tree (Hedges et al., 2006). All birth‐and‐death events of *Tas2rs* were placed in each branch of the species tree to show the evolutionary trajectories of *Tas2r* repertoires in Amphibia.

### 2.5 Adaptive evolution analysis of *Tas1r* genes

The selective pressure of *Tas1r* genes was tested by two steps. First, the number of nonsynonymous substitutions per nonsynonymous site (d*N*) and the number of synonymous substitutions per synonymous site (d*S*) were used to compute overall ω (d*N*/d*S*) values. *ω* = 1, *ω* < 1, and *ω* > 1 represent neutral, purifying, and positive selection, respectively. The mean ω for each *Tas1r* was calculated by the CodeML method (Yang, 2007) with EasyCodeML (Gao et al., 2019). The generally accepted phylogenetic tree was inferred from TimeTree (Hedges et al., 2006). Moreover, positive selection could act on individual amino acid residue. Therefore, in the second step, codon‐based analyses with CodeML and FUBAR (Murrell et al., 2013) were performed to detect potential positive selection sites. In CodeML, the models between M7 (purifying selection) with M8 (positive selection) with EasyCodeML were compared. A likelihood ratio test was used to estimate whether there was a significant difference between the models. FUBAR analysis on the Datamonkey server (http://classic.datamonkey.org/; Pond et al., 2005) was used to find evidence of episodic positive/diversifying selection with a posterior probability of 0.9.

## 3 RESULTS

### 3.1 *Tas1r* and *Tas2r* repertoires

This study annotated 16 *Tas1r1*, 9 *Tas1r2*, and 9 *Tas1r3* intact sequences that appear to be functional genes (Table 1). One truncated *Tas1r1* and *Tas1r2* in common frog and one truncated *Tas1r3* in Tibetan Plateau frog were also identified as pseudogenes (see Appendix 2 for the genomic location). We failed to identify the *Tas1r1* gene from genome assemblies of the western clawed frog and the African clawed frog. Thus, synteny analysis was performed to examine whether *Tas1r* is lost or not. *Tas1r1* is flanked by *NOL9* and *ZBTB48*. This linearity is conserved across human, mouse, and two‐lined caecilian. The presence of *NOL9* and *ZBTB48* next to *Tas1r1* was confirmed, providing evidence of whole *Tas1r1* deletion in the two taxa (Appendix 3). Genes that flank *Tas1r2* in mice and most species surveyed (*MIB2*, *GOLIM4*) were located on the same contig in American bullfrog, Tibetan Plateau frog, African clawed frog, western clawed frog, and Gaboon caecilian. In the genome of the two‐lined caecilian, *Tas1r3* is flanked by *DVL1* and *CPTP*. *DVL1* and *CPTP* were adjacent to each other on the same contig in Leishan spiny toad, African bullfrog, American bullfrog, strawberry poison frog, African clawed frog, and western clawed frog (Appendix 3). Thus, it was speculated that perhaps the *Tas1r2*/*Tas1r3* gene is lost in the respective species. Hence, the absence could lead to the inactivation of both umami and sweet taste functions in Leishan spiny toad, African bullfrog, American bullfrog, strawberry poison frog, Asiatic toad, western clawed frog, and African clawed frog and the loss of sweet taste function in Tibetan Plateau frog and tiny cayenne caecilian (Table 1). In addition, multiple copies of *Tas1r1* genes in five amphibian genomes were found. Nevertheless, duplication of *Tas1r2* only occurred in the common frog, and duplication of *Tas1r3* only occurred in common frog, Tibetan Plateau frog, and tiny cayenne caecilian.

**TABLE 1:** Summary of *Tas1r* gene family and functional prediction of umami/sweet taste in amphibian

Name	Species	Order	*Tas1r1*	*Tas1r2*	*Tas1r3*	Umami (Tas1r1‐Tas1r3)	Sweet (Tas1r2‐Tas1r3)
Leishan spiny toad	*Leptobrachium leishanense*	Anura	1	1	0	×	×
Mexican spadefoot toad	*Spea multiplicata*	Anura	2	1	1	√	√
African bullfrog	*Pyxicephalus adspersus*	Anura	2	1	0	×	×
American bullfrog	*Lithobates catesbeianus*	Anura	2	0	0	×	×
Common frog	*Rana temporaria*	Anura	2(1PS)	2(1PS)	2	√	√
Tibetan Plateau frog	*Nanorana parkeri*	Anura	2	0	2 (1PS)	√	×
Eastern banjo frog	*Limnodynastes dumerilii*	Anura	1	1	1	√	√
Strawberry poison frog	*Oophaga pumilio*	Anura	1	1	0	×	×
Asiatic toad	*Bufo gargarizans*	Anura	1	1	0	×	×
African clawed frog	*Xenopus laevis*	Anura	0	0	0	×	×
Western clawed frog	*Xenopus tropicalis*	Anura	0	0	0	×	×
Two‐lined caecilian	*Rhinatrema bivittatum*	Gymnophiona	1	1	1	√	√
Gaboon caecilian	*Geotrypetes seraphini*	Gymnophiona	1	0	1	√	×
Tiny cayenne caecilian	*Microcaecilia unicolor*	Gymnophiona	1	1	2	√	√

Note

√, putative function; ×, putative disfunction.

Abbreviation: PS, Pseudogene.

**FIGURE 1:**
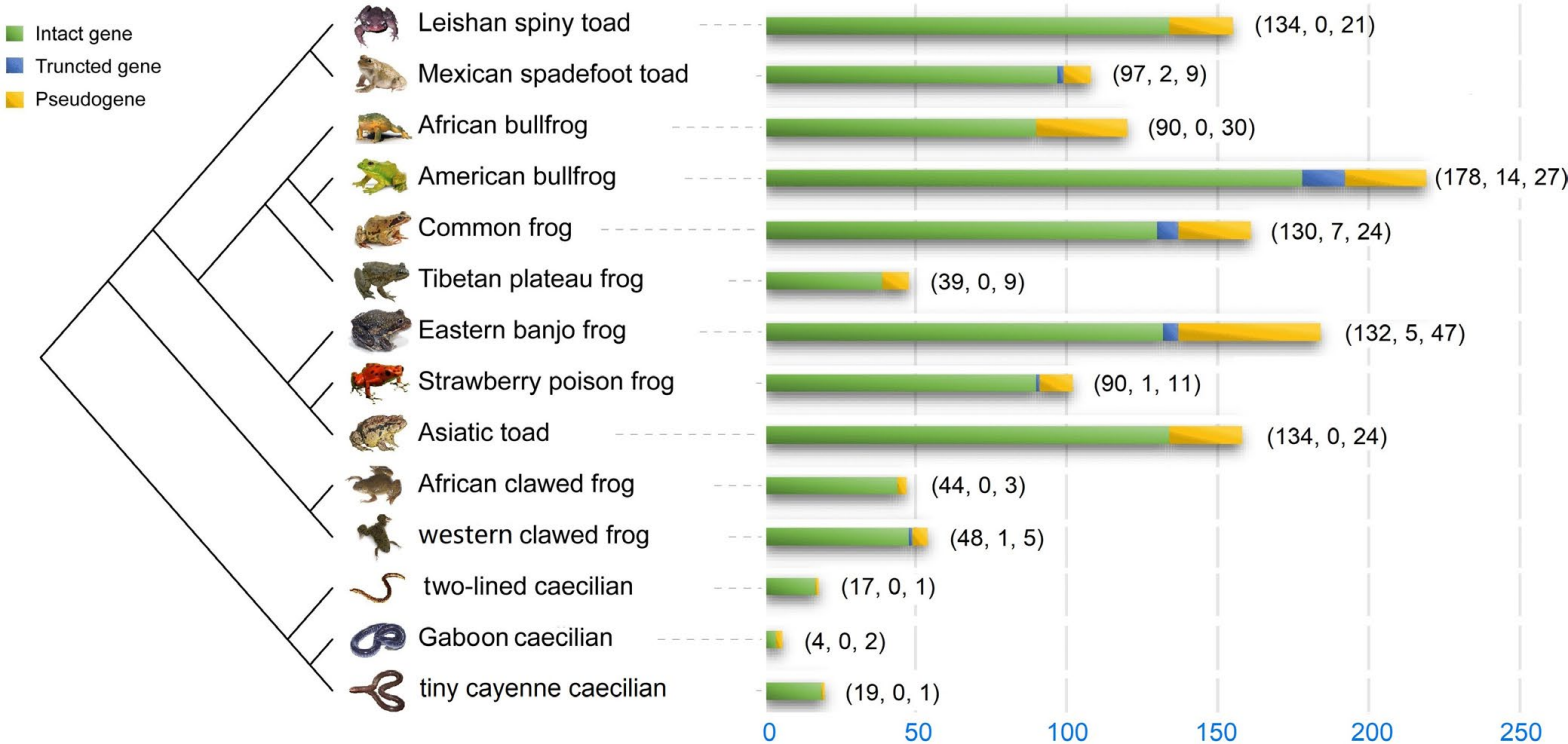
Numbers of intact, partial, and pseudo *Tas2r* genes in 14 amphibian species. The numbers in brackets denote intact genes, partial genes, and pseudogenes, respectively

This study annotated 1400 *Tas2r* genes in amphibian genome assemblies, including 1156 intact, 30 partial, and 214 pseudo *Tas2rs* (Figure 1). The genomic locations of each *Tas2r* gene are uploaded in Zenodo (https://doi.org/10.5281/zenodo.5642517). Each species in Gymnophiona possessed a medium‐sized *Tas2r* repertoire, ranging from 4 to 19. In sharp contrast, the *Tas2rs* number in order Anura was from 44 to 178. The American bullfrog presented the largest number of *Tas2r* genes (*n *= 178) not only in amphibians but also for any species investigated so far. The nucleotide length of intact genes was 816–1272 bp, with an average of 942 bp. The number of partial genes was from 0 to 14. The number and the percentage of pseudogenes ranged from 1 to 47 and from 5.0% to 33.3%, respectively. Overall, it showed a considerable variance in the number of intact genes between the two orders in amphibians. The *Tas2r* gene repertoire size in Anura species was much larger than in not only Gymnophiona but also other vertebrate groups.

To detect whether tandem duplication happens in amphibians, the genomic location of *Tas2r* genes was examined. Indeed, some *Tas2rs* were organized into clusters on specific chromosomes or scaffolds (Appendix 4). For example, *Tas2r* genes were mainly located on chromosomes 4, 6, 7, and 13 in Leishan spiny toad, chromosomes 4 and 7 in African bullfrog, and chromosomes 4, 8, and 11 in the Asiatic toad. Interestingly, *Tas1rs* also occurred in neighboring intergenic regions. For instance, multiple copies of the *Tas1r1* gene of Mexican spadefoot toad, African bullfrog, common frog, and Tibetan Plateau frog were located in the same chromosome or scaffold of each species. Similar results were also found in *Tas1r3* (Appendix 2). These results suggested that tandem duplications of *Tas1rs*/*Tas2rs* could be one cause of the expansion of the two gene families.

### 3.2 Phylogeny of *Tas1r* and *Tas2r* genes

**FIGURE 2:**
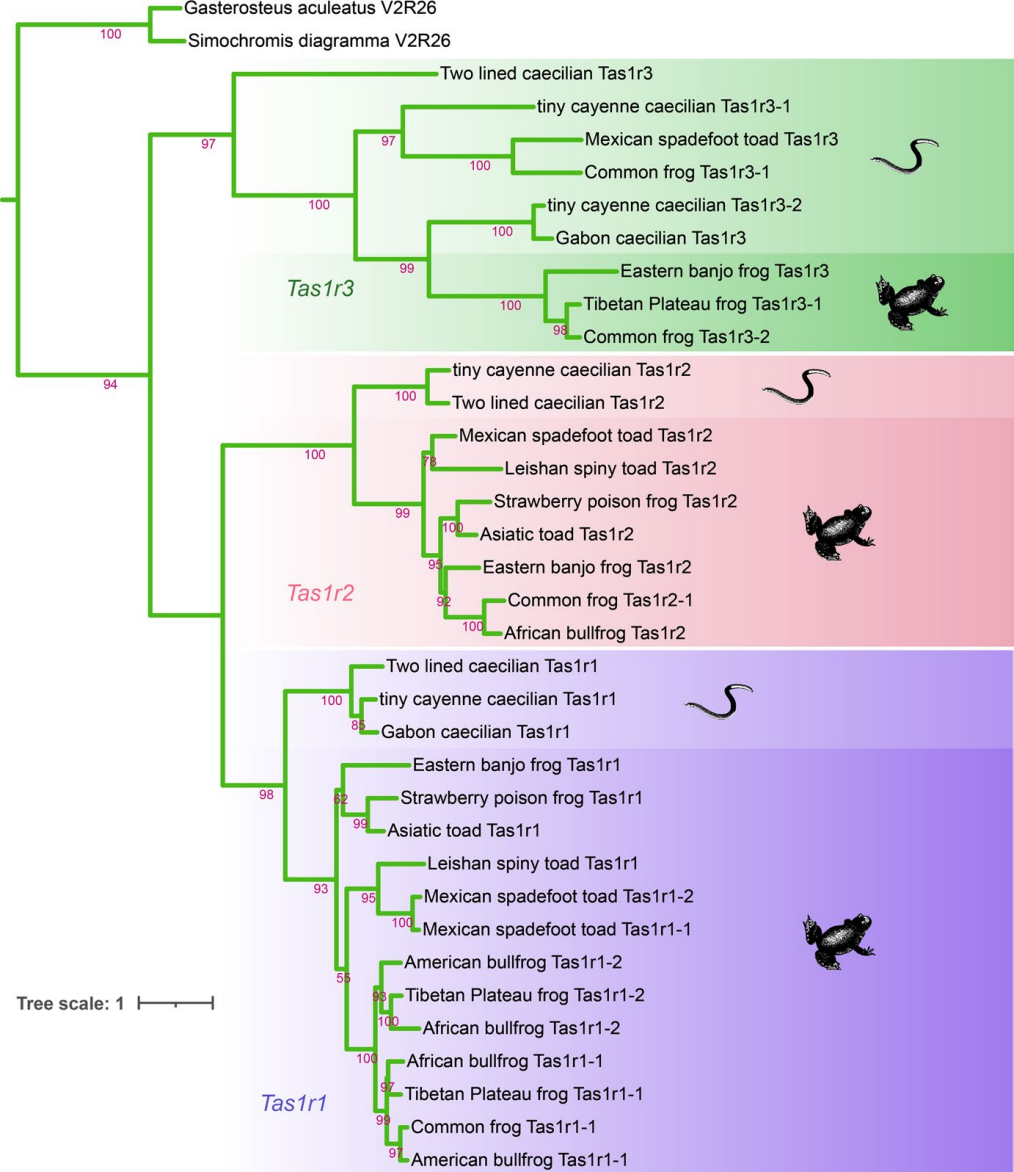
Evolutionary relationships of intact *Tas1r* genes in amphibians. The phylogenetic tree was constructed using the ML method. Phylogeny was rooted with the vomeronasal 2 receptor 26 gene (*V2R26*) of two fish species *Gasterosteus aculeatus* (NCBI accession no. XM_040193080.1) and *Simochromis diagramma* (NCBI accession no. XM_040009575.1). This is because *V2R* genes are relatively close to *Tas1r* genes among GPCRs. The numbers at the branches indicate the percentage of posterior probability values

To delineate the evolutionary history and relationships among amphibian *Tas1r*/*Tas2r* genes, phylogenetic analysis by the ML method based on all intact genes was performed. Figure 2 shows the phylogenetic relationship of intact *Tas1r* genes. Most branches in the tree showed high bootstrap support, indicating the reliability of phylogenetic relationships among *Tas1rs*. Clusters of *Tas1r1*, *Tas1r2*, and *Tas1r3* could separate from each other. Each gene cluster formed into two clades, including anurans and caecilians.

**FIGURE 3:**
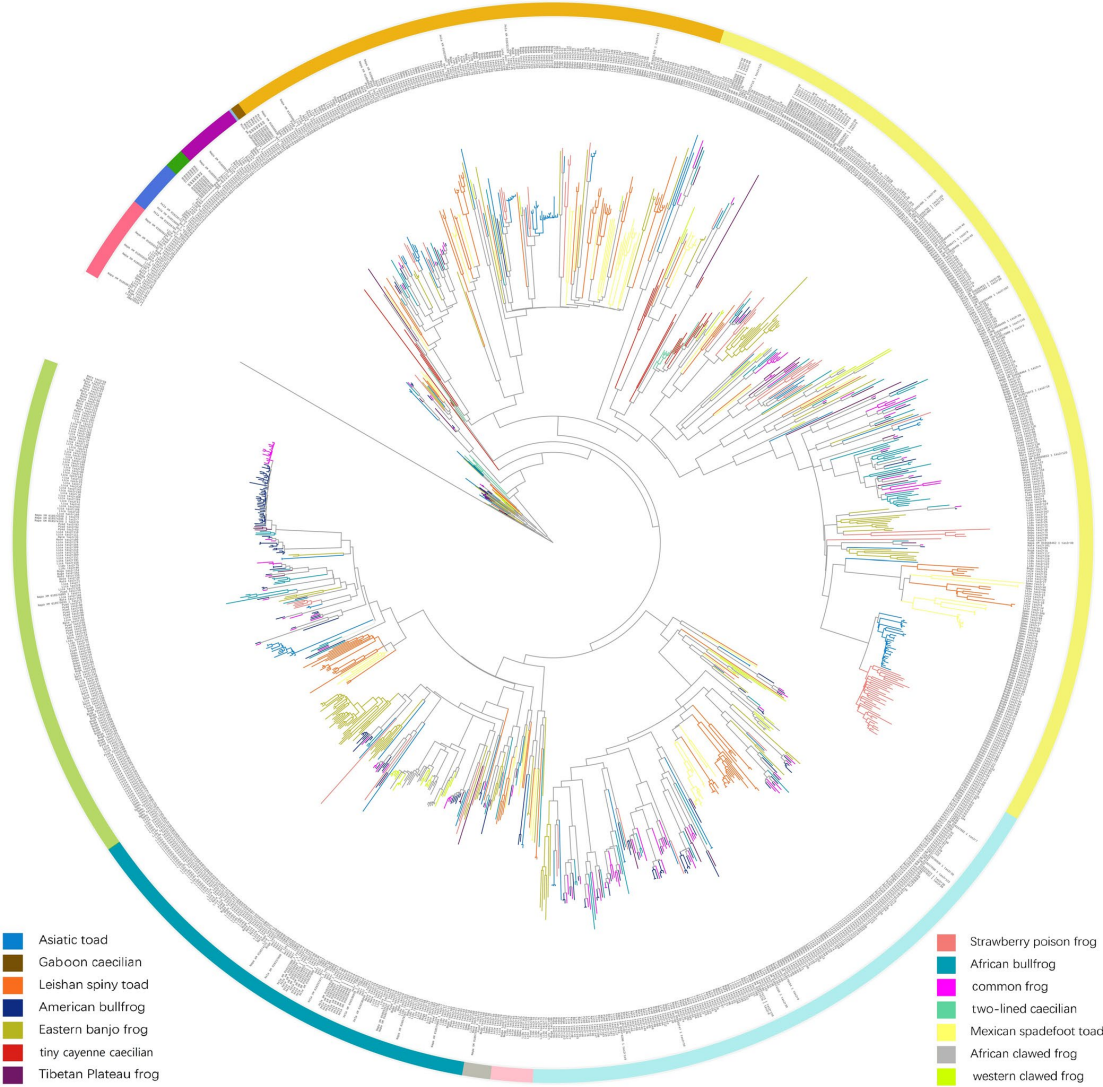
Evolutionary relationships of 1156 intact *Tas2r* genes in amphibians. The phylogenetic tree was constructed using the ML method. The vomeronasal 1 receptor 3 gene (*V1R3*; NCBI accession no. AB670529) of East African cichlids *(Lithochromis xanthopteryx*) was used to root the tree because *V1R* genes are relatively close to *Tas2r* genes among GPCRs. Genes from different species are indicated by different colors of branches

Based on the phylogenetic tree, intact *Tas2r* genes were categorized into five large and eight small clades (Figure 3). Some lineages showed a cluster of *Tas2r* genes from the same species (marked with one color), suggesting that these lineages are enriched with species‐specific gene duplications. In contrast, other lineages showed genes from distantly related species.

### 3.3 Lineage‐specific gene births and deaths of *Tas2rs*

**FIGURE 4:**
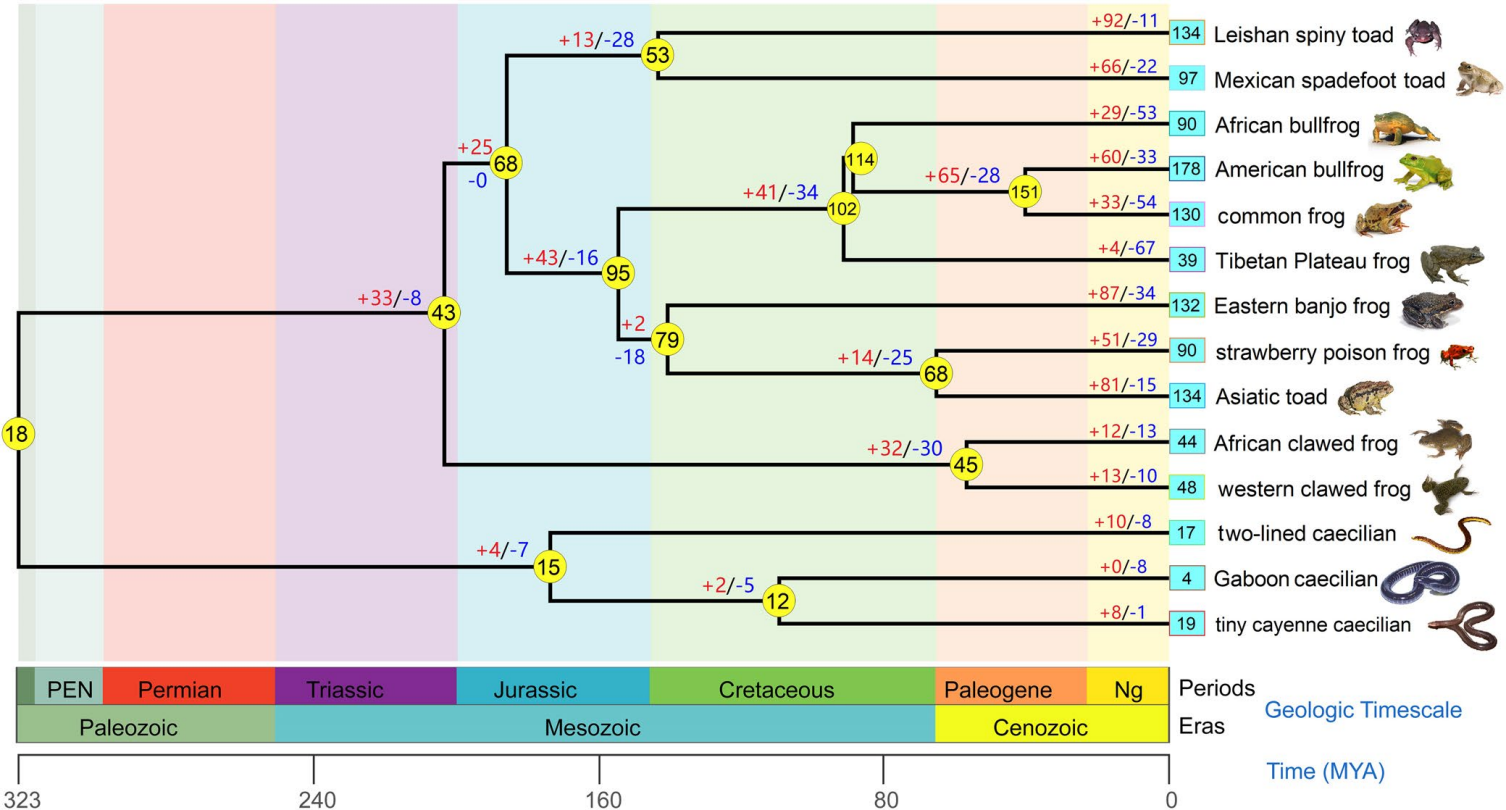
Evolutionary trajectories of amphibian *Tas2r* gene repertoires. The numbers in circles and boxes denote the number of intact *Tas2rs*. The numbers on branches denote gene increases (+; caused by gene duplication) and decreases (−; caused by gene deletion). For example, Leishan spiny toad gained 92 *Tas2rs* and lost 11 *Tas2rs* after branching off from its common ancestor with the Mexican spadefoot toad. The phylogenetic relationships and divergence times of these species were referred to TimeTree (Hedges et al., 2006)

The phylogeny of *Tas2r* genes implies that extensive gene expansions may have occurred in the Anura lineage or the contractions took place in the Gymnophiona lineage. To infer the evolutionary changes of *Tas2r* numbers in amphibians, a reconciliation analysis was performed by comparing the gene tree and species tree. Results showed that total duplications and losses were 851 and 555, respectively (Figure 4). Overall, frequent and dramatic gene birth and death events occurred in almost each branch. Conservatively, results indicated that the common ancestor of amphibians had at least 18 intact *Tas2rs*. After a gene gain (*n* = 33) and loss (*n *= 8) in the ancestral lineage of Anura, the *Tas2rs* number of the common ancestor of Anura increased to 43. Moreover, further reductions (−7, −5) were observed in the branch of Gymnophiona. Because the increase occurred in Anura (43 intact *Tas2rs*) compared to Gymnophiona (15 intact *Tas2rs*), data suggested that the reduction of *Tas2rs* may have occurred before the divergence between Anura and Gymnophiona. As shown in the evolutionary trajectory tree, extensive species‐specific gene duplications may be responsible for the considerably larger *Tas2r* repertoires in some anurans, for instance, 92 gains in Leishan spiny toad and 81 gains in the Asiatic toad (Figure 4).

### 3.4 Purifying selection in the *Tas1r* gene family

To understand the selective pressure of the *Tas1r* gene family, the ratio (d*N*/d*S*) of nonsynonymous mutation rate (d*N*) to synonymous mutation rate (d*S*) was calculated. All *Tas1r* genes were under strong purifying selection (0.169–0.210; Table 2). This result indicated that *Tas1rs* were evolving‐constrained, and the function was conserved in the species, which remained as the relevant *Tas1r* genes. A small number of positively selected sites were found in each gene interspecies.

**TABLE 2:** Selection Analysis of Amphibian *Tas1rs*

Gene	Number of sequences^a^	aa Length	d*N*/d*S*	Positively selected sites^b^	Negatively selected sites^b^
*Tas1r1*	16	557	0.169	3	428
*Tas1r2*	9	450	0.187	7	302
*Tas1r3*	9	620	0.210	6	369

^a^ Number of sequences for each *Tas1r* gene.

^b^ Positively/negatively selected sites were detected with CodeML (M7/M8) and FUBAR methods (posterior probability = 0.9).

## 4 DISCUSSION

Studies on umami, sweet, and bitter taste receptors have made enormous progress in recent years. Despite the special evolutionary status of amphibians, their taste receptor families have been rarely described except for the western clawed frog (Behrens et al., 2014; Go, 2006; Shi & Zhang, 2006). In this study, the repertoire of *Tas1r* and *Tas2r* genes from a wide collection of amphibian species was presented for the first time. Unlike the conservation of only one copy of *Tas1r1*/*Tas1r2*/*Tas1r3* in numerous vertebrates (Shi & Zhang, 2006), two copies of the *Tas1r* gene in several amphibian species were found. Similarly, duplication events of the *Tas1r* gene (*n* = 1–4) were also reported in several teleost (Dong et al., 2018). The result may support the fact that amphibians diverge from the ancestral fish‐tetrapod stock during the evolution of animals from strictly aquatic forms to terrestrial types. This study confirmed previous findings and showed that all three *Tas1rs* were absent in the western clawed frog (Shi & Zhang, 2006). Note that the annotations of the *Tas1r* gene in the genome database are sometimes incorrect. For example, *V2R* genes of the African clawed frog and the western clawed frog were annotated as *Tas1r1* in the NCBI probably due to the sequential similarity of the two GPCR families. To ensure our prediction accuracy, annotated genes were verified in the genome database with reciprocal BLAST, synteny analysis, and phylogenetic analyses.

Sweet and umami tastes help animals to recognize dietary information for nutritious carbohydrates and proteins, respectively, and thus are pivotal for the survival of animals. However, given the similarity of feeding preferences in amphibians and their distinct phylogenetic positions, this study failed to discover a correlation between feeding ecology and *Tas1r* evolution. Most of the dietary preference of anurans are similar to each other, but their *Tas1r* genes can be intact, pseudogenized, or absent, suggesting that no correlation exists between *Tas1r* functionality and feeding ecology. It seemed that loss of umami/sweet tastes could occur in any species. This study found that *Tas1r1* genes of the African clawed frog are absent from its genome assembly. Surprisingly, robust glossopharyngeal nerve responses have been recorded in amphibians when various amino acids are applied to taste organs on the tongue (Feder & Burggren, 1992; Gordon & Caprio, 1985; McPheeters & Roper, 1985; Yoshii et al., 1982). The African clawed frog has been reported to have high gustatory sensitivity to amino acid, such as arginine (0.1–1.0 μM; Yoshii et al., 1982). The above contradiction between the absence of *Tas1r1* genes and amino acid sensitivity could be explained by the following evidence. Although Tas1r1+3 functions as the main umami receptor in mammals, non‐*Tas1r1* genes responsible for detecting amino acids likely exist. For example, an odorant receptor, preferentially tuned to recognize basic amino acids, was identified in goldfish (Speca et al., 1999). The odorant receptor shares sequence similarities with calcium sensing, metabotropic glutamate, and V2R vomeronasal receptors (Speca et al., 1999). To the authors’ knowledge, the western clawed frog has the largest V2R repertoire in 14 species investigated (Urszula Brykczynska et al., 2013; Chen et al., 2019; Shi & Zhang, 2007). It was supposed that a mass of V2Rs may be involved in detecting amino acids. Analysis of gustatory nerve responses in metabotropic glutamate receptor 4 (mGluR4) knockout mice provided functional evidence for the involvement of mGluR4 in umami taste responses (Yasumatsu et al., 2014). As for sweet taste, research on gustatory transduction in taste cells demonstrated that the ability to detect sweet substances is present in frogs (Kusano & Sato, 1958; Toshihide et al., 1995). The cAMP or cGMP cascade may be involved in the transduction of sweet stimuli in bullfrog TRCs (Kolesnikov & Margolskee, 1995). It would be interesting to examine whether there are other transduction mechanisms involved in the umami/sweet sensation of amphibians.

This study reported the largest *Tas2rs* family for any species so far. Meanwhile, the number of *Tas2rs* genes (especially intact genes) varies greatly among different amphibian species. Although the African clawed frog, derived from the diploid species western clawed frog, has undergone a whole‐genome duplication (WGD) event to be a tetraploid species, it possesses a moderate size of *Tas1r*/*Tas2r* repertoire compared to other amphibians. This finding suggests that WGD may not have played a major role in the evolution of the amphibian *Tas1r*/*Tas2r* repertoire.

A large repertoire of *Tas2rs* in amphibians likely reflects their adaptation to variable lifestyles and environments. Most frogs and toads inhabit both aquatic and terrestrial habitats, which necessarily contain a larger variety of toxic substances. Accordingly, their ecological needs should encompass vastly different requirements for their taste system. For instance, aquatic factors, such as pH were proven to have influenced the divergence of taste receptor genes (Caprio et al., 2014; Lin et al., 2004). A great number of *Tas2rs* could also fulfill their dietary needs. Although some larger amphibian species eat vertebrates, most frogs feed on worms, insects, and other small arthropods that contain more potentially toxic substances.

Aside from environmental selections, amphibians’ dietary preferences may change. On the one hand, the diet of some anurans tends to vary ontogenetically. For instance, the metamorphic transition from aquatic larvae to terrestrial adults imposes dietary shifts. Larval anurans are almost exclusively microphagous herbivores or detritivores (Altig et al., 2007; Duellman & Trueb, 1986; Montaa et al., 2019; Wassersug & Heyer, 1988). After metamorphosis, most anurans become insectivores (Duellman & Trueb, 1986). Plant‐eating frogs are scant in the literature and include only *Bufo marinus*, *Bufo regularis*, *Rana esculenta*, and *Rana hexadactyla* (Da Silva & De Britto‐Pereira, 2006). Interestingly, the hylid frog *Xenohyla truncata* is unique among frogs with its frugivorous feeding biology (Da Silva et al., 1989; Da Silva & De Britto‐Pereira, 2006). Among these diets, plant materials are rich in bitter substances (Glendinning, 1994; Wang et al., 2004), and insects can secrete defensive poisonous chemicals (always tastes bitter) to deter predators (Howse, 1975). Hence, bitter tasting compounds should have strongly affected the diversity of the *Tas2rs* repertoire in herbivorous and insectivorous amphibians. On the other hand, diet tends to vary seasonally along with prey availability (Donnelly, 1991). As anurans are primarily visual, opportunistic predators (sit‐and‐wait foraging), their selection of prey is limited by the gape of the predator. As a result, the biological importance of bitter taste likely resides in the ability to detect and reject unpalatable, potentially dangerous prey once it is captured, rather than detecting prey (Barlow, 1998). Only after visually selected prey have reached the mouth, does the taste system function as a toxin detector such that unpalatable and potentially poisonous food is spat out. Hence, we speculate that the lifestyle, ecological, and dietary complexity of amphibians elevate their evolutionary pressure for a wide variety of *Tas2r* genes. The large number of *Tas2rs* is consistent with anatomical evidence showing more taste buds and a larger number of taste receptors in amphibians than other vertebrates (Kinnamon & Cummings, 1992; Kinnamon & Margolskee, 1996; Lindemann, 1996). Terrestrial and aquatic anurans have enlarged, specialized organized taste disks that often are found atop large epithelial papillae (Barlow, 1998; Reutter & Witt, 1993), perhaps contributing to specific adaptations for tasting in air and water. Moreover, large numbers of *Tas2rs* may be answered by the exquisite sensitivity and tuning properties in amphibians. Behrens et al. detected the tuning breadth of six Tas2rs of the western clawed frog with 46 bitter compounds. Their results showed that three Tas2rs recognize numerous agonists, whereas the other three Tas2rs are narrowly tuned. That said, a large Tas2r repertoire of the western clawed frog may allow the development of specialized receptors, possibly for toxins with species‐specific relevance (Behrens et al., 2014).

Caecilians are highly adapted for a burrowing existence. They primarily dwell in highly organic, friable surface layers of the soil, where they maintain tunnel systems. As far as is known, all caecilians are carnivores. Free‐ranging diet includes earthworms, platyhelminths, arthropods, frog eggs, tadpoles, and anoline lizards (Bogert, 1970; Daniel, 1998; Wake, 1994). Because animal tissues contain fewer toxic chemicals, it implies reduced importance of bitter taste in caecilians compared with anurans.

In general, this study characterized *Tas1r*/*Tas2r* genes and investigated their evolution. It will not only provide abundant raw data but also further recover the evolution dynamics of *Tas1r*/*Tas2r* genes. In particular, studying these genes in the large‐scale evolutionary unit can reflect the evolutionary process more comprehensively and systematically. It will also help provide accurate data support for research on function and feeding behavior.

## CONFLICT OF INTERESTS

The authors declare that they have no competing interests.

## AUTHOR CONTRIBUTIONS

**Huaming Zhong:** Conceptualization (lead); Data curation (lead); Methodology (lead). **Jie Huang:** Data curation (equal); Methodology (equal). **Shuai Shang:** Data curation (equal); Methodology (equal). **Baodong Yuan:** Funding acquisition (lead); Project administration (lead).

## Data Availability

The genomic locations or GenBank accessions of *Tas2r* genes are available at Zenodo (https://doi.org/10.5281/zenodo.5642517).

## APPENDIX 1

Whole‐genome assemblies analyzed in the present study

**Table:**

Name	Species	Family	Prefix^a^	Assembly	N50c^b^	Genome coverage (×)
Leishan spiny toad	*Leptobrachium leishanense*	Megophryidae	Lele	GCA_009667805.1	1,946,319	80.3
Mexican spadefoot toad	*Spea multiplicata*	Pelobatidae	Spmu	GCA_009364415.1	30,692	21
African bullfrog	*Pyxicephalus adspersus*	Pyxicephalidae	Pyad	GCA_004786255.1	30,445	189
American bullfrog	*Lithobates catesbeianus*	Ranidae	Lica	GCA_002284835.2	5,415	66
Common frog	*Rana temporaria*		Rate	aRanTem1.1	2,889	68
Tibetan Plateau frog	*Nanorana parkeri*	Dicroglossidae	Napa	GCA_000935625.1	32,798	83
Eastern banjo frog	*Limnodynastes dumerilii*	Limnodynastidae	Lidu	GCA_011038615.1	10,550	156
Strawberry poison frog	*Oophaga pumilio*	Dendrobatidae	Oopu	GCA_009801035.1	5,836	136
Asiatic toad	*Bufo gargarizans*	Bufonidae	Buga	GCA_014858855.1	1,738,317	103
African clawed frog	*Xenopus laevis*	Pipidae	Xela	GCA_001663975.1	19,713	30
Western clawed frog	*Xenopus tropicalis*		Xetr	GCA_000004195.4	14,634,335	111.5
Two‐lined caecilian	*Rhinatrema bivittatum*	Rhinatrematidae	Rhbi	GCA_901001135.1	3,216,284	43
Gaboon caecilian	*Geotrypetes seraphini*	Dermophiidae	Gese	GCA_902459495.1	20,656,571	67
Tiny cayenne caecilian	*Microcaecilia unicolor*	Siphonopidae	Miun	GCA_901765105.1	3,661,507	53

^a^ The prefix is used to discriminate the name of orthologs in species. For example, *Leptobrachium leishanense Tas2r1* is referred to as Lele*Tas2r1*.

^b^ N50c is the contig length where 50% of the assembled genome lies in blocks of at least N50C.

## APPENDIX 2

The GenBank accession numbers or genomic locations of the *Tas1r* genes in this study

**Table:**

Species	*Tas1r1*	*Tas1r2*	*Tas1r3*
Leishan spiny toad (*Leptobrachium leishanense*)	CM019073.1: 49019172–49026858	CM019073.1: 22996556–23009832	No BLAST results
Mexican spadefoot toad (*Spea multiplicate*)	*Tas1r1*‐*1*: VKOC01000007.1: 19288589–19294065 *Tas1r1*‐*2*: VKOC01000007.1: 19294512–19300129	VKOC01000007.1: 14768290 14774206	VKOC01000007.1: 14776162–14790438
African bullfrog (*Pyxicephalus adspersus*)	*Tas1r1*‐*1*: CM016426.1: 53245503–53258080 *Tas1r1*‐*2*: CM016426.1: 53263053–53272308	CM016426.1: 4957042–44966384	No BLAST results
American bullfrog (*Lithobates catesbeianus*)	*Tas1r1*‐*1*: KV949322.1: 15569–39104 *Tas1r1*‐*2*: KV954354.1: 16846–37639	No BLAST results	No BLAST results
Common frog (*Rana temporaria*)	*Tas1r1*‐*1*: Chr10: XM_040326700.1 *Tas1r1*‐*2_PS*: Chr10: XM_040326949.1	*Tas1r2*‐*1*: Chr10: XM_040326656.1 *Tas1r2_PS*: Chr10: XM_040326657.1	*Tas1r3*‐*1*: Chr10: XM_040325817.1 *Tas1r3‐2* Chr10: XM_040325818.1
Tibetan Plateau frog (*Nanorana parkeri*)	*Tas1r1*‐*1*: NW_017306273.1: XM_018576608.1 *Tas1r1*‐*2*: NW_017306273.1: XM_018576630.1	No BLAST results	*Tas1r3*‐*1*: NW_017307970.1: XM_018571215.1 *Tas1r3*‐*2_PS*: NW_017307970.1: XM_018571216.1
Eastern banjo frog (*Limnodynastes dumerilii*)	WWET01001477.1: 12319–39339	WWET01002568.1: 114560–128901	WWET01002568.1: 130002–161269
Strawberry poison frog (*Oophaga pumilio*)	VIAB01020550.1: 24836–46751	VIAB01072821.1: 390–4617+VIAB01059777.1: 1–5576	No BLAST results
Asiatic toad (*Bufo gargarizans*)	CM026466.1: 628630042–628648059	CM026466.1: 599424218–599444854	No BLAST results
African clawed frog (*Xenopus laevis*)	No BLAST results	No BLAST results	No BLAST results
Western clawed frog (*Xenopus tropicalis*)	No BLAST results	No BLAST results	No BLAST results
Two‐lined caecilian (*Rhinatrema bivittatum*)	Chr15: XM_029578055.1	Chr15: XM_029579313.1	Chr15: XM_029578066.1
Gaboon caecilian (*Geotrypetes seraphini*)	Chr15: XM_033921681.1	No BLAST results	Chr15: XM_033921380.1
Tiny cayenne caecilian (*Microcaecilia unicolor*)	Chr13: XM_030222159.1	Chr13: XM_030185735.1	*Tas1r3*‐*1*: Chr13: XM_030222148.1 *Tas1r3*‐*2*: Chr13: XM_030222149.1

## APPENDIX 3

Scaffold and gene location of the flanking genes. The genome of two‐lined caecilian was used as a reference, in which the neighboring genes of *Tas1r1* are *Nol9* and *Zbtb48*, *Tas1r2* are *MIB2* and *Golim4*, and in *Tas1r3*, they are *Dvl1* and *Cptp*

**Table:**

Species	Gene	Scaffold	Flanking gene/position subject	Flanking gene/position subject
	***Tas1r1***		***Nol9* (702aa)**	***Zbtb48* (688aa)**
African clawed frog		NC_054383.1	103990021–104013200	104132287–104149606
Western clawed frog		NC_030683.2	99469716–99486727	99583487–99595828
	***Tas1r2***		***Mib2* (972aa)**	***Golim4* (681aa)**
Tibetan Plateau frog	–	NW_017307970.1	35880–89028	272720–316421
African clawed frog	–	NC_054384.1	81727385–81795251	81649520–81701317
Western clawed frog	–	NC_030683.2	92207281–92280709	92329059–92379641
Gaboon caecilian		NC_047098.1	20095591–20181939	20224665–20270222
	***Tas1r3***		***Dvl1* (695aa)**	***Cptp* (216aa)**
Leishan spiny toad	–	KN616455.1	94046–107939	4890–56539
African bullfrog		PZQJ01000011.1	*45518180 −45584538*	*45466313–45503990*
Strawberry poison frog	–	LVCR01025734.1	92–4420	10784–12869
African clawed frog	–	KN628019.1	106433–139608	97714–99564
Western clawed frog	–	LD637301.1	281024–324221	330574–332913

## APPENDIX 4

*Tas2rs* were organized into clusters on specific chromosomes or scaffolds
